# Revealing the Genetic Components Responsible for the Unique Photosynthetic Stem Capability of the Wild Almond *Prunus arabica* (Olivier) Meikle

**DOI:** 10.3389/fpls.2021.779970

**Published:** 2021-11-25

**Authors:** Hillel Brukental, Adi Doron-Faigenboim, Irit Bar-Ya’akov, Rotem Harel-Beja, Ziv Attia, Tamar Azoulay-Shemer, Doron Holland

**Affiliations:** ^1^Unit of Fruit Tree Sciences, Institute of Plant Sciences, Agricultural Research Organization, Newe Ya’ar Research Center, Ramat Yishay, Israel; ^2^The Robert H. Smith Institute of Plant Sciences and Genetics in Agriculture, Faculty of Agriculture, Hebrew University of Jerusalem, Rehovot, Israel; ^3^Department of Vegetable and Field Crops, Institute of Plant Sciences, Agricultural Research Organization, Volcani Center, Rishon Lezion, Israel

**Keywords:** almond, *Prunus arabica*, wild almond, stem photosynthesis, QTL, genetic mapping, deciduous fruit trees

## Abstract

Almond [*Prunus dulcis* (Mill.) D. A. Webb] is a major deciduous fruit tree crop worldwide. During dormancy, under warmer temperatures and inadequate chilling hours, the plant metabolic activity increases and may lead to carbohydrate deficiency. *Prunus arabica* (Olivier) Meikle is a bushy wild almond species known for its green, unbarked stem, which stays green even during the dormancy period. Our study revealed that *P. arabica* green stems assimilate significantly high rates of CO_2_ during the winter as compared to *P. dulcis* cv. Um el Fahem (U.E.F.) and may improve carbohydrate status throughout dormancy. To uncover the genetic inheritance and mechanism behind the *P. arabica* stem photosynthetic capability (SPC), a segregated F1 population was generated by crossing *P. arabica* to U.E.F. Both parent’s whole genome was sequenced, and SNP calling identified 4,887 informative SNPs for genotyping. A robust genetic map for U.E.F. and *P. arabica* was constructed (971 and 571 markers, respectively). QTL mapping and association study for the SPC phenotype revealed major QTL [log of odd (LOD) = 20.8] on chromosome 7 and another minor but significant QTL on chromosome 1 (LOD = 3.9). As expected, the *P. arabica* allele in the current loci significantly increased the SPC phenotype. Finally, a list of 64 candidate genes was generated. This work sets the stage for future research to investigate the mechanism regulating the SPC trait, how it affects the tree’s physiology, and its importance for breeding new cultivars better adapted to high winter temperatures.

## Introduction

Almond, *Prunus dulcis* (Mill.) D. A. Webb, is a major fruit tree crop worldwide. As a deciduous fruit tree, it enters dormancy during early winter and renews growth following the fulfillment of a variety-specific period of exposure to low temperatures, known as chilling requirements (CR) and adequate heat requirements. Exposure to a sufficient number of low winter temperatures is essential for synchronized flowering in the early spring followed by efficient pollination, fruit set, and fruit development (Sánchez-Pérez et al., 2014). CR limit growing areas of deciduous fruit trees and dramatically influence the yield and quality of fruit (Atkinson et al., 2013). When winter temperature increases, CR are not sufficiently provided, and the metabolic activity increases (Sperling et al., 2017). As a result, carbohydrates are consumed, and intense starch synthesis occurs. These changes lead to soluble carbohydrate (SC) deficiency in the buds during the period of flowering and fruit set, which results in disruptive flowering that may reduce the yield (Tixier et al., 2017; Fernandez et al., 2018; Guo et al., 2021). The ability of the dormant almond to respond this energy depletion is restricted, mainly due to the shortage in photosynthetic leaves during dormancy. Climate-changing trends emphasize the urgent need for deciduous fruit crops to gain more plasticity (Gradziel et al., 2001), particularly for maintaining their non-structural carbohydrate (NSC) reserves in warmer winters (Atkinson et al., 2013; Zwieniecki et al., 2015).

**FIGURE 1:**
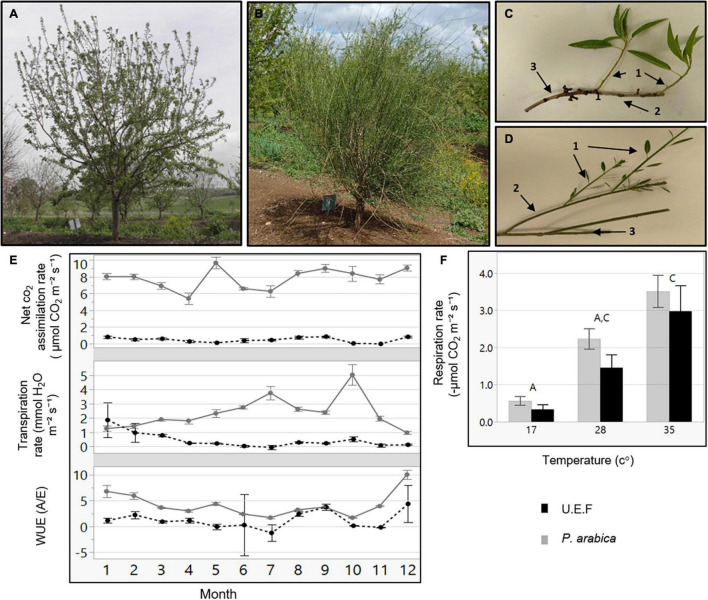
Gas exchange measurements of *P. arabica* and *Prunus dulcis* cv. Um el Fachem (U.E.F.) stems. Three-year-old trees of the Israeli cultivar *Prunus dulcis* cv. U.E.F. **(A)**, and the wild almond *P. arabica* **(B)** at spring. Stems of U.E.F. **(C)**, and *P. arabica* **(D)** annually developed: 1-year-old (c1 and d1), 2-year (c2 and d2), and 3-year-old stems (c3 and d3). Gas exchange data of 1-year-old stems along the year **(E)** of *P. arabica* (solid gray line), and U.E.F. (dashed black line). Each dot denotes the average of two independent days of measuring for each month (*n* = 8). Stem respiration rate in response to three different temperatures **(F)** of *P. arabica* (gray bar) and U.E.F. (black bar). Four stems were measured for each genotype in each temperature (*n* = 4). Different capital letter represents significance (α = 0.05) between temperatures, not between species. The error bars represent ± SE.

*Prunus arabica* (Olivier) Meikle, also known as *Amygdalus arabica* Olivier, is defined as a different species from the domesticated almond *P. dulcis*. However, both belong to the *Prunus* genus and are a part of the Rosacea family. The species “arabica” was named after the geographical region where it was first described. This taxon is native to the temperate-Asia zone. It covers the Fertile Crescent Mountains, Turkey, Iran, and Iraq. In the Middle East, it can be found in Lebanon, Syria, Israel (Judean Desert), and Jordan (Roskov et al., 2019). *P. arabica* can be found in altitudes between 150 and 1,200 m and rarely up to 2,700 m. It is a bush, rather than a tree, with a very long root system and is considered resistant to drought (Sorkheh et al., 2011; Rajabpoor et al., 2014). As a deciduous tree, *P. arabica* drops its leaves at the end of the summer, turns meristems into buds, and stops growing. However, unlike other almond species, its young branches remain green and are not covered with bark (i.e., no cork layer deposition) throughout the dormancy phase (Figures 1A–D). In fact, *P. arabica* stems remain moist and green during the whole year. *P. arabica* green stems were previously suggested to photosynthesize (Sorkheh et al., 2009), yet no physiological evidence was published regarding their ability to assimilate external CO_2_.

Stem photosynthesis was previously shown in other desert species (Aschan and Pfanz, 2003). In these species, which do not belong to the Rosacea family, high efficiency of CO_2_ assimilation comparable with that of the leaf was demonstrated. Because stem photosynthesis was found in desert plants, it was suggested that it might play a role in carbon gain under stress conditions such as heat and drought (Nilsen, 1995). The contribution of stem photosynthesis to tree adaptation under drought was further supported by evidence showing that stem photosynthesis assists in embolism repair (Bloemen et al., 2016; De Baerdemaeker et al., 2017). The ability to photosynthesize through stems could prove to be highly beneficial for deciduous fruit trees such as almonds in maintaining the energy balance of the tree, particularly during hot winters and springs when respiration is enhanced, and tree energy is limited due to leaf drop and the lack of photosynthetic organs (Zwieniecki et al., 2015; Sperling et al., 2019). Previous genetic studies of *P. arabica* were limited to phylogenetic studies and encompass a small number of markers (few to dozens) (Yazbek and Oh, 2013; Delplancke et al., 2016). To the best of our knowledge, no genetic approach that uncovered the mechanism of the stem photosynthesis phenomena was reported.

Recent important advancement in Rosacea genetics and genomics enables the application of a genetic approach in the study of important physiological processes. Such advancements include the development of genetic maps based on F1 and F2 populations (Sánchez-Pérez et al., 2014) and their usage for mapping QTLs affecting CR in apple (*Malus domestica*) (Miotto et al., 2019), pear (*Pyrus communis*) (Gabay et al., 2018), apricot (*Prunus armeniaca)* (Olukolu et al., 2009), and peach (*Prunus persica)* (Wang et al., 2002). In almonds, genetic mapping of hybrid populations based on distinctive CR demonstrated that a major gene *LATE BLOOMING* (*LB*) was associated with blooming date and dormancy release (Ballester et al., 2001). In addition, complete genomes and various transcriptomic datasets of Rosacea, apple, cherry (*Prunus avium*), and peach (Jung et al., 2019), were published. Recently, two almond genomes were published: *P. dulcis* cv. Texas^1^ and *P. dulcis* cv. Lauranne.^2^ This trend of new genomic and transcriptomic data of deciduous fruit trees, including almonds, sets the stage for intense genetic research and the development of novel marker-assisted breeding approaches. In this report, we combined physiological and genetic approaches to study the green stems of *P. arabica* throughout the year. By direct measurements of gas exchange, we demonstrate that *P. arabica* stems transpire and assimilate significant levels of CO_2_ all-year-round, including during the dormancy period. Moreover, we undertook a forward genetic approach and used an F1 population segregating for the stem photosynthesis trait for high-resolution mapping of major QTLs for this trait. The genetic markers and candidate genes that our study underlines pave the way to undermine the physiological role of stem photosynthesis and its utilization for genetic improvement.

## Results

### *Prunus arabica* Assimilate CO_2_ Through Green Stems

*Prunus arabica* stems remain green during winter, while cultivated almonds develop an outer gray cork layer (Figures 1B,D). To study if these stems are actively assimilating CO_2_, we undertook gas exchange measurements of tree stems in the orchard with the Licor 6800 Portable Photosynthesis System. Two different almond species were compared, namely, the wild almond *P. arabica* and the cultivated almond *P. dulcis* (U.E.F.), throughout the entire year (Figure 1E). The data indicate that *P. arabica* assimilated CO_2_ through its green stems during all year (annual average of 8 ± 0.19 μmol CO_2_ m^–2^ s^–1^), while similar 1-year-old stems of U.E.F. assimilation capacity is almost null (annual average of 0.5 ± 0.05 μmol CO_2_ m^–2^ s^–1^). The significantly high CO_2_ assimilation rates of *P. arabica* stems were found comparable with assimilation rates of *P. arabica* leaves (11.2 ± 0.8 CO_2_ m^–2^ s^–1^, July average, data not shown). Although some fluctuations observed between the different seasons, pronounced high CO_2_ assimilation rates were found in *P. arabica* stem during the whole year (Figure 1E). Finally, *P. arabica* stem transpiration rate is relatively low in the dormancy phase and gradually increases until it peaks in October (1.2 ± 0.18 in January to 5 ± 0.7 mmol H_2_O m^–2^ s^–1^ in October). In contrast, transpiration from U.E.F. stems is relatively constant and low throughout the year (0.46 ± 0.11 mmol H_2_O m^–2^ s^–1^; Figure 1E). Transpiration rate fluctuation is also attributed to high instantaneous water-use efficiency (iWUE) of *P. arabica* during the dormancy phase (two fold higher than U.E.F. in December; Figure 1E).

Previous studies on temperate fruit trees showed a positive correlation between tissue temperature and SC consumption through respiration (Sperling et al., 2019). To find out how stem respiration of *P. arabica* and U.E.F. are influenced by temperature, we measured the respiration rate of 1-year-old stems while exposing them to three different temperatures (17°, 28°, and 34°C) (Figure 1F). Increased respiration rate in response to elevated temperature was observed in both almond species (0.5 ± 0.11, 2.2 ± 0.27, 3.5 ± 0.44, and 0.33 ± 0.12, 1.4 ± 0.35, 3 ± 0.68 μmol CO_2_ m^–2^ s^–1^ for *P. arabica* and U.E.F., respectively, for each temperature), while no significant differences were observed between species (for each measured temperature).

### Stem Assimilation Is Genetically Inherited

**FIGURE 2:**
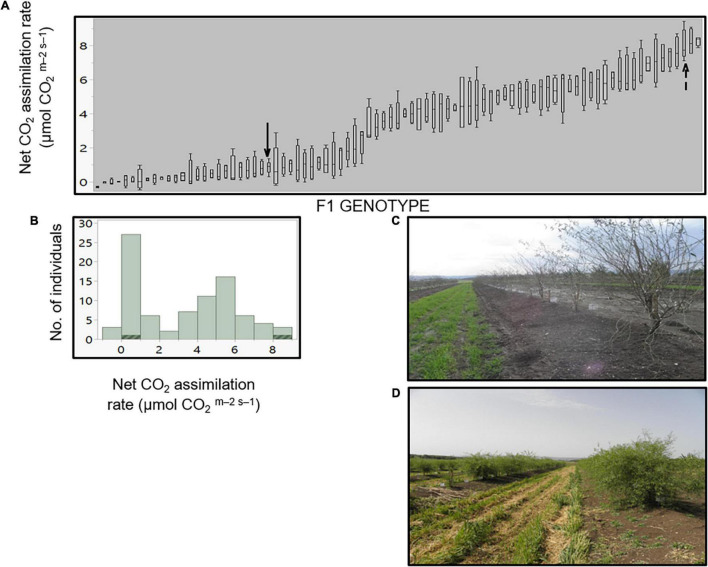
Stem photosynthetic capability (SPC) in the F1 progeny. Levels of net CO_2_ assimilation for each offspring **(A)**. Each box plot presents the average of four stems (*n* = 4). The F1 progeny parents, *P. arabica* and U.E.F. are marked by dashed arrow and simple arrow, respectively. Distribution histogram of the same data is presented in **(B)**, while parents’ data are highlighted. Measurements were conducted during February 2020, while the trees were dormant. Representative pictures of the F1 population while dormant **(C)** in February, and during the vegetative phase in April **(D)**.

To elucidate the genetic nature of the assimilating stem trait of *P. arabica*, an F1 hybrid population (*n* = 92) was established by crossing *P. arabica* (male) with U.E.F. (female) (Figures 2C,D). The same approach of gas exchange measurements in the field was used for phenotyping the SPC trait among the 3-year-old F1 population during dormancy. Twelve offspring assimilated CO_2_ *via* their stems in a similar level as *P. arabica* (offspring 24H27 is the highest; 8.3 ± 0.14 μmol CO_2_ m^–2^ s^–1^), and 37 individuals assimilated as U.E.F. or less, all other offspring phenotypes fluctuated between the parents (Figure 2A). Analysis of distribution demonstrated two prominent peaks within the histogram (Figure 2B). “3 Normal Mixture” is the most accurate model to describe the current phenotype distribution (achieved the lowest AICc and the −2 log-likelihood values). Broad-sense heritability (h^2^) was found to be high (0.91).

### Sequence Comparisons, SNPs Identification, and Genotyping of the F1 Population

Segregation of the SPC trait rendered the F1 population as suitable infrastructure for genetic mapping. For this purpose, we sequenced the *P. arabica* and the U.E.F. genomic DNA, targeting for high coverage, to ensure reliable (SNP) calling. The reads were aligned against the reference genome of *P. dulcis* cv. Lauranne, because better mapping results were obtained with the Lauranne genome as compared to *P. dulcis* cv. Texas genome (> 97% and ∼85%, respectively) (Table 1).

**TABLE 1:** Quality data from whole-genome sequencing of *P. arabica* and U.E.F.

Species	Average coverage	Q20	Q30	% Mapping vs. Lauranne	% Mapping vs. Texas	Total variant sites	% Heterozygous variant sites
*P. arabica*	∼ X 57	94.5	87.37	97.93	85.94	3,750,363	35.18
U.E.F. *(P. dulcis)*	∼ X 55.5	94.7	87.71	98.37	89.82	2,407,787	69.29

*Quality parameters (Q20 and Q30) from sequencing of each parent are presented with respect to the reference genomes of *P. dulcis* cv. Lauranne and *P. dulcis* cv. Texas. The row sequence data were mapped to each of the reference genomes. The total variant sites (SNPs and InDels) between each species and cv. Lauranne reference genome are also presented.*

A total of 3,750,363 and 2,407,787 variants (i.e., SNPs or short InDels) were detected for *P. arabica* and U.E.F., respectively, against the cv. Lauranne reference genome. Analyzing the variants showed that 71.5 and 72.6% (*P. arabica* and U.E.F., respectively) are in the intergenic region (Supplementary Table 1). Furthermore, a higher variant number was detected in the intronic regions in relation to the exons (Supplementary Table 1). The initial number of identified SNPs (total variant sites in Table 1) was filtered by several types of criteria as specified in section “Materials and Methods.”

Overall, 4,887 SNPs that are heterozygous for one of the parents and homozygous for the second were selected for F1 genotyping screening. The SNPs are spread at intervals of about 40K along the almond genome.

**FIGURE 3:**
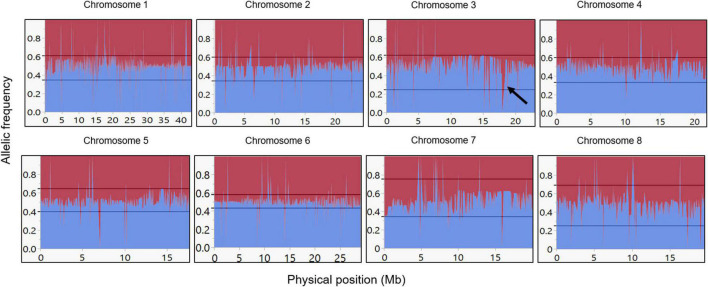
Allelic frequency among the F1 population. Allelic frequency of the heterozygous allele in the F1 population for each marker. Blue line indicates the *P. arabica* allelic incidence, and red line indicates the U.E.F. allelic incidence. *X*-axis is the physical position in Mb, and *y*-axis represents the allelic ratio (from 0 to 1). Black arrow in chromosome 3 represents an example of unexpected deviated region (“hot spot”). References lines presented the “tolerance interval” limits.

The F1 population was successfully genotyped with 4,612 SNPs. The resulting genotyping quality data (Supplementary Table 2) represent high coverage (152X) and low number of missing data (5.5%). Further analysis of the genotyped F1 population with the SNP panel described above shows that the allelic frequency within the F1 population is 50%, as expected from an F1 population (Figure 3). Moreover, this expected ratio suggests that there is no plant contamination in our F1 plant material. However, since the allelic composition in this bi-parental population is AA × Aa, we can also refer this ratio as the allelic frequency of the heterozygous genotype. Therefore, data presented (Figure 3) also indicate exceptional chromosomal regions (hot spot) with a unique pattern of inheritance that deviates from the 1:1 ratio, for example, in chromosome 3 (see black arrow in Figure 3).

### Construction of Genetic Maps for the F1 Population

**FIGURE 4:**
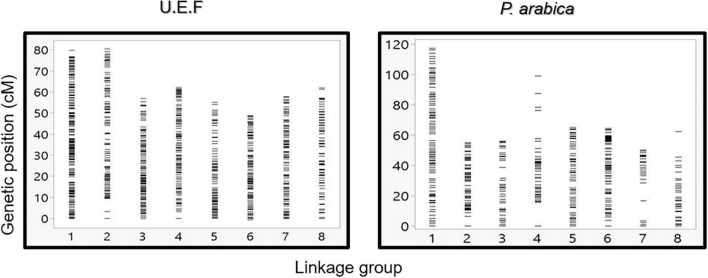
Graphic presentation of markers density and distribution along the eight linkage groups. Comparison between U.E.F. map (left graph) and *P. arabica* map (right graph). Each horizontal line represents a single marker.

To establish a genetic map of the F1 population, Join Map 4.1 software was used (Van Ooijen and Jansen, 2013). CP (cross-pollination) population type was performed with the lmxll code for markers that were homozygous for the male parents (*P. arabica*) and heterozygous for the female parent (U.E.F.). The code nnxnp was used for the opposite case. A significant portion of the markers was filtered; most of them were due to complete similarity (∼50%). Overall, 1,533 SNPs were used for mapping (Table 2). Because there were no common markers for both parents, the hkxhk code was not applied. Using the pseudo-test cross-method (Olukolu et al., 2009), we separated the markers for two different maps: one map for the U.E.F. (where *P. arabica* is homozygous, lmxll code), and the second map for the *P. arabica* parent (where U.E.F. is homozygous, nnxnp code). Applying this strategy, we obtained two maps with robust numbers of markers and good density. The U.E.F. map was found to be denser than the *P. arabica* map and includes 971 markers with an average distance of 0.533 centiMorgan (cM), while *P. arabica* map contains 572 SNPs with an average distance of 1.093 (cM) (Table 2). It can be clearly seen that the distribution of the SNP markers is well spread (the biggest gap is 6.173 cM in the U.E.F. and 18.85 cM in *P. arabica* map) over the eight almond linkage groups (LGs) (Figure 4).

**TABLE 2:** Characteristics of the established genetic maps.

Map type			Number of markers	LGs number	Total length (cM)	Marker density (cM)			
		
	U.E.F.	*P. arabica*				Average	Median	Maximum distance	SE
1	Aa	aa	971	8	504.64	0.533	0.375	6.173	0.017
2	AA	Aa	572	8	568.76	1.093	0.768	18.854	0.078

*U.E.F. map (1) was generated from markers that were homozygous in *P. arabica* (male) and heterozygous in the U.E.F. (female). *P. arabica* map (2) was generated from markers that were homozygous for U.E.F. and heterozygous for *P. arabica*.*

**FIGURE 5:**
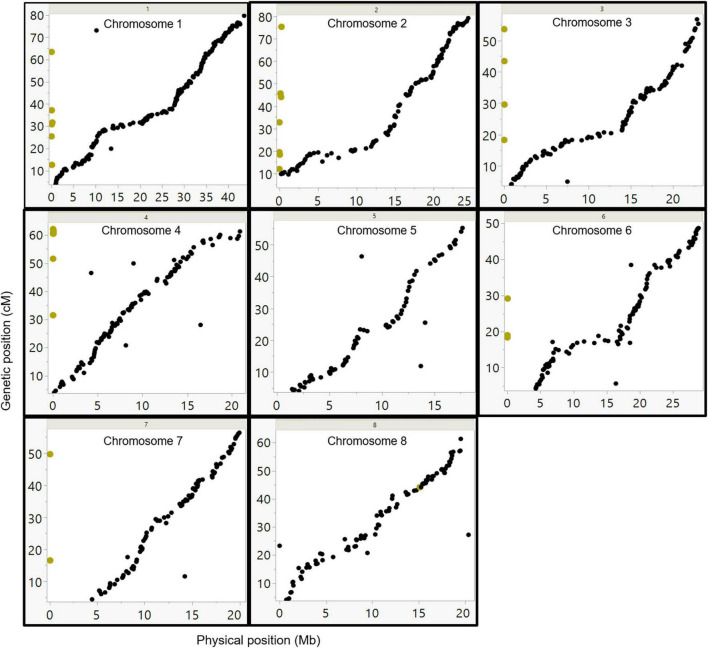
Comparison between the genetic and physical order of the markers. SNP markers were placed according to their physical position on the Lauranne reference genome sequence (*x*-axis), and their position on the U.E.F. genetic map (*y*-axes). The yellow dots represent the markers from unplaced scaffolds, according to the Lauranne reference genome (chr-0). Those markers were mapped to several chromosomes in this study based on the genetic map data.

To assess the validity of the genetic map, the order of SNP markers as determined by the U.E.F. genetic map was compared with the deduced order from the physical map as determined by cv. Lauranne reference genome. The analysis (Figure 5) demonstrates a good colinearity between the genetic and the physical map. Remarkably, most of the markers from the genetic map were highly correlated with the physical order (Figure 5), yet, few markers did not correlate (chromosome 6; Figure 5). The genetic map divided the markers into eight LGs parallel to the previously published chromosome organization order. Moreover, the slopes generated between the physical orders to the genetic order represent recombination frequency (cM/Mb). Thus, one can see that around the centromere, the slope is more horizontal, meaning the cM/Mb ratio is relatively low. Thirty-eight SNP markers representing un-scaffold contigs (i.e., chromosome 0) in the reference genome project were assembled into six LGs based on the genetic maps (marked by yellow dots in Figure 5).

### QTL Analysis and Genome-Wide Association Study of the Stem Photosynthetic Capability Trait

**FIGURE 6:**
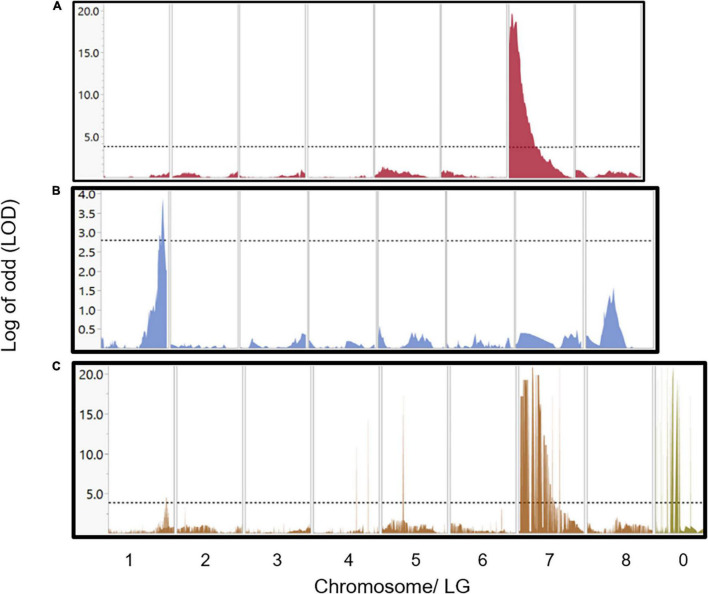
QTLs and GWAS analysis for the SPC trait. Major QTL of 2.4 cm width and LOD score of 20.8 was detected in chromosome 7 by using the U.E.F. genetic map **(A)**. Minor QTL of 4.4 cm width and LOD score (3.9) was located at the end of chromosome 1 by using the *P. arabica* genetic map **(B)**. Results of GWAS using the whole set of markers (3,800) sorted by their physical position according to the reference genome **(C)**, revealed both loci in chromosome 7 and chromosome 1. Markers in c were sorted by their physical position according to the reference genome. Markers that were placed on chromosome 0 and found as highly associated with the SPC trait are also shown in **(C)**. The horizontal dash line represents the significance level according to permutation test (1,000 times at α = 0.05).

Two main approaches were initiated for detecting genomic regions regulating the stem photosynthetic capability (SPC). QTL mapping, computed with Map QTL by interval mapping (IM) analysis (Van Ooijen, 2006) and genome-wide association study (GWAS) by TASSEL software (Bradbury et al., 2007). QTL mapping generated two significant QTLs. Each QTL was discovered only in one of the two genetic maps. Thus, one major QTL (LOD = 20.8) was mapped on LG 7 spanning a region of 2.4 cM detected on the U.E.F. map. The second, minor but significant QTL (LOD = 3.9) was detected at the end LG 1, spanning a region of 4.4 cM on the *P. arabica* map (Supplementary Table 3 and Figure 6).

Applying GWAS approach with TASSEL enabled us to simultaneously detect two genomic sites that regulate the SPC on chromosomes 1 and 7 at positions similar to those detected by QTL mapping. The major region on chromosome 7 spans only 400 kb, and the minor on chromosome 1 contains 700 kb. Moreover, GWAS analysis showed significant associations with markers aligned to chromosome 0. Interestingly, two of these markers assembled into the major QTL in locus 7 (Supplementary Table 3, marked with gray background). The major QTL explained 67% of the phenotypic variance, while the minor QTL explained 19.3% (Supplementary Table 3).

### Interaction of QTLs

**FIGURE 7:**
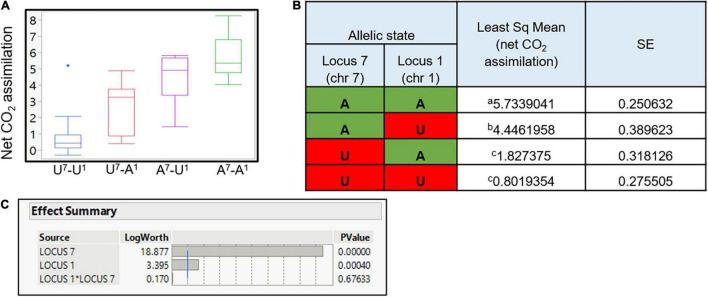
QTLs effect on net CO_2_ assimilation and the synergistic effect between QTLs. Least square of means for each allelic combination is presented **(A)**. Each box plot represents the population individual’s average phenotype grouped for their allelic combination. *Y*-axis is the level of net CO_2_ assimilation. The *x*-axis represents the allelic combination. On the *x*-axis, the capital letter A refers to individuals with *P. arabica* allele combination, U refers to individuals with U.E.F. allele combination in each one of the QTL, and superscript numbers (7 and 1) present the QTL identity. Numerical presentation of the data is presented in **(B)**. *P. arabica* allele combination is marked in green, and U.E.F. marker combination is marked in red. Different letters indicate significance (α = 0.05). Statistical evaluation of each QTL effect, and interaction between the two loci **(C)**. Presented results were analyzed by the “full factorial test”; blue line is equivalent for *P*-value of α = 0.01.

As presented, two significant loci were discovered as regulating the SPC (Figure 6 and Supplementary Table 3). Full factorial test shows a significant additive effect between those two associated loci (*P* < 0.0001; Figures 7A,B). Yet, no epistatic effect was found (*P*-value = 0.676; Figure 7C). As expected, in both QTLs, the *P. arabica* alleles were the increasing alleles regarding the SPC trait.

### Generating a List of Candidate Genes

Combining the data on the QTLs bordering markers [which were wider than the one obtained by the associated region detected in GWAS (Supplementary Table 3)] with that of the cv. Lauranne reference sequence allowed us to delineate a list of genes within the regions that are predicted as responsible for the SPC trait. In total, 350 genes were detected under the QTLs (Supplementary Table 5). The region at chromosome 1 includes 82 annotated genes with SNPs between *P. arabica* and U.E.F. Among those, 15 include non-synonymous SNPs in the gene-coding region. The associated region at chromosome 7 consists of 218 genes with SNPs, of which only 49 have non-synonymous SNPs in their coding region. The data are based on the usage of the SnpEff software (Cingolani et al., 2012; Supplementary Table 1) with the NCBI genome database (GCA_008632915.2).

## Discussion

### *Prunus arabica* Can Assimilate CO_2_ Through Its Green Stems All-Year-Round

This study investigated the wild almond *P. arabica*, a member of the Rosacea family. We demonstrated for the first time that the wild almond *P. arabica* is assimilating external CO_2_ *via* its green stems all-year-round, including during the dormancy period when the tree sheds its foliage. In contrast, the cultivated almond U.E.F. is unable to do so (Figure 1E). Interestingly, even the young green stems of U.E.F. (Figure 1C), which are not covered with bark layer, in spring, are assimilating CO_2_ in a negligible levels (Figure 1E). *P. arabica* is a desert plant, and its native growing ecosystems encompass the desert margins of the Fertile Crescent (Roskov et al., 2019). Previous studies have indicated that perennial desert shrubs, such as *Ambrosia salsola* and *Bebbia juncea*, are able to photosynthesize through their stems (Ávila-Lovera et al., 2019). This capability was attributed to better adaptation of the plant to arid climate conditions (Aschan and Pfanz, 2003). The geographical distribution of *P. arabica* suggests that stem photosynthesis might be important for its survival in dry areas (Nilsen, 1995). Indeed, several studies have suggested that stem CO_2_ assimilation may help prevent embolism damage in response to drought (Bloemen et al., 2016; De Baerdemaeker et al., 2017). Others have claimed that SPC enables more efficient carbon gain with respect to water loss (transpiration) due to smaller surface area when compared to leaves (Ávila-Lovera et al., 2017). Interestingly, fluctuations were observed in transpiration rates of *P. arabica* stem during the year, which resulted in maximal iWUE in winter time since CO_2_ assimilation was not highly affected (Figure 1E). Further physiological and anatomical analyses are needed to understand better this unique SPC trait and its contribution to almonds in harsh climates.

Among the wild and cultivated species of almond, only *P. arabica* and its very close relative, *Prunus scoparia* (Spach) Schneider, are known to produce all-year green stems (Khadivi-Khub and Anjam, 2014). Yet stem CO_2_ assimilation in *P. scoparia* was not demonstrated. To our knowledge, *P. arabica* is the only known deciduous fruit tree that possesses the SPC trait. As such, *P. arabica* is unique among all deciduous fruit trees in its capability to assimilate external CO_2_ from stems in winter. In addition to the reasonable assumption that SPC contributes to plant survival in dry conditions by preventing embolism damage and by improving carbon gain, it is anticipated that SPC in winter could influence other critical physiological processes that are dependent on carbohydrate management, including dormancy break and ability to support heavy yield (Granot et al., 2013; Guo et al., 2021). This is particularly true for deciduous fruit trees under the pressure of energy shortage when the trees have to support both leaf and flower development (Fernandez et al., 2018). Deciduous fruit trees rely on their energy reserves during this growth phase since their leaves are not yet fully developed (Sperling et al., 2019). Recent studies on deciduous fruit trees demonstrate how higher temperatures are positively correlated with tree NSC consumption. It was shown that during higher winter temperatures, NSC reserves depleted rapidly (Zwieniecki et al., 2015). For setting these results in *P. arabica* and U.E.F., we measured respiration rate in response to elevated temperatures. As expected, environmental temperature indeed raised the respiration rate in both species (Figure 1F). This result emphasizes the possible link between winter temperatures and trees’ NSC status during winter. Importantly, it highlights the advantage of a functional photosynthetic organ in warm winters during dormancy phase. Nevertheless, to establish the link between tissue temperature to tree NSC and their contribution to yield, we are currently conducting additional research.

### The Stem Photosynthetic Capability Trait Depicts a High Heritability Value and Segregates in an F1 Population

As a means of investigating the functional role of SPC in almonds, we undertook a forward genetics approach. Identification of the genetic components underlying SPC is crucial for deciphering the role of SPC in respect to dormancy break and adaptation to upcoming climate changes. Moreover, molecular markers for the genes in question could provide an efficient tool for breeding and genetic manipulation for better almond trees. For this purpose, we established an F1 population from a cross between *P. arabica* and U.E.F. Remarkably, SPC measurements of individual progenies within the F1 population indicate that the SPC trait is inherited and segregated already in the F1 population (Figure 2A). Interestingly, the distribution of the trait is not normal but displays two main peaks, suggesting that this trait is probably controlled by a small number of genes or QTLs (Figure 2B). Altogether, the data indicated that the F1 hybrid population could be used as a mapping population for the SPC trait. Furthermore, the high heritability value (0.91) suggesting the SPC trait can be integrated into cultivars through classical genetic crosses. Yet, current data are not sufficient to determine the heritability control of the trait (dominant/recessive) due to the continuous nature of the SPC phenotype.

### Genotyping

In this study, we report the feasibility of using a novel methodology to obtain genetic markers through QTL mapping of almond. The method of “targeted SNP seq” that was already described for other plants (Zhang et al., 2020) takes advantage of the availability of almond chromosomal organization and genome sequencing data. Only markers that are evenly spread along the chromosomes at an average of 40K were chosen in order to target the desired marker density and genomic distribution. High coverage for parent’s whole-genome sequencing (WGS), and the targeted genotyping, yielded a good SNPs panel with almost no missing data or out-filtered SNPs for low quality.

The expected segregation of the selected markers (i.e., the parent’s alleles) is 1:1, meaning the allelic frequency should be 0.5 on average. Indeed, our results, which established the genotyping quality of the SNPs in the F1 population, match this prospect (Figure 3). The allelic frequency by physical position presentation shows a small number of “hot spots” that display genomic regions with an unexpected segregation ratio (see black arrow on chromosome 3; Figure 3), where the frequency of the parent’s allele is significantly deviating from 1:1 ratio. This may indicate that the SNPs in that region could be placed in or near a lethal allele, which its presence in the progeny is not favorable. Such an allele, for example, could be an incompatibility *S* allele (Gómez et al., 2019).

### Genetic Maps Construction

Two maps were constructed, each for a different parent (Figure 5). The map for the U.E.F. was denser, with 971 markers divided for all the LGs (Table 2 and Figure 5). Both maps were fitted to the eight chromosomes published in the two almond reference genomes (text foot notes 1, 2). The current U.E.F. map density (one marker per ∼0.5 cM) is almost equal to the average recombination frequency (0.475 cM per Mb) (Table 2). Considering this, together with the small population size (*n* = 92), we assume that the recombination rate (i.e., population size) and not the number of markers is the bottleneck for obtaining better mapping resolution.

High co-linearity between the physical and the genetic maps (Figure 5) demonstrates the reliability of the constructed map. Furthermore, it gives an overview of the genomic distance between the wild almond species, *P. arabica*, and *P. dulcis* cultivar cv. Lauranne, which, remarkably, seems to be quite similar. Moreover, 98% of the sequenced reads of *P. arabica* were mapped to cv. Lauranne reference genome. Although two reference genomes are available for almond, a genetic map is essential for two main reasons. First, genetic map, which relies on recombination frequency of the current population, should represent the most accurate result for marker arrangement of the population compared to the reference genome (Oren et al., 2020). In this respect, both genetic maps conform well to the physical data of the cv. Lauranne reference genome. Nonetheless, the few markers that are not correlating could emphasize some chromosomal aberrations as translocation. Second, using the genetic map, 38 markers that were delineated as chromosome 0 were linked to LGs by recombination (see yellow dots in Figure 5).

The F1 population and the character of markers selected resulted in the construction of two maps. SNP markers are mainly di-alleles; by choosing SNPs that are heterozygous for one parent and homozygous for the other, we can assure that the markers will segregate and also identify the parental origin of each allele. In order to intersect the maps, shared markers are needed. Joining the F1 maps could be achieved by SSR markers that have more than di-alleles, as was done in apricot F1 population (Hurtado et al., 2002), or by SNPs that are heterozygous for both parents, as was done in pear (Gabay et al., 2018).

### Stem Photosynthetic Capability Genetic Mapping

Linkage analysis (i.e., QTL mapping) and GWAS were two approaches used in this study for linking the SPC phenotype and genetic markers (Figure 6 and Supplementary Table 3). While GWAS only associates between genomic markers without any data on their specific genomic location, the linkage analysis, which is based on a genetic map, connects phenotype to a specific genomic locus/loci (Oren et al., 2020).

Mapping of QTL discovered two significant loci. One major locus with an exceptional LOD score of ∼20 on LG/chromosome 7 was found in the U.E.F. map, and another minor (LOD score of ∼4) was detected at the end of LG/chromosome 1 in *P. arabica* map. GWAS revealed both loci. Moreover, the loci identified by the GWAS overlapped those found by QTL analysis. Six markers on chromosome 0 also demonstrate significant LOD score (∼20) (Figure 6 and Supplementary Table 3). Using the genetic map and the QTL analysis enabled the positioning of two of these markers to the QTL of locus 7, while the other markers were positioned in other LGs, or discarded during the genetic map construction procedure (Supplementary Table 3). Here, we demonstrated the importance of integrating the association data, which is based on the physical reference genome and the QTL mapping approach based on the F1 recombination frequency. The GWAS enabled us to run the analysis with higher number of SNPs comparing to the genetic map (3,700 vs. 970 in the U.E.F. genetic map). The overlapping results between the two methods emphasize the unbiased genetic infrastructure and corroborate the mapping data. The data presented identified new genetic loci on the almond genome. To our knowledge, such a mapping effort on the SPC trait has never been done before for any plant, above all, in trees. These strong mapping data are important in order to fully comprehend the physiological role of SPC and identify new genetic components that control photosynthesis in plants. The availability of segregating population and genetic markers highly associated with SPC provides a powerful means to explore this trait.

### Candidate Genes

The high-resolution QTL mapping and the robust annotations data sets available enabled the establishment of a putative list of candidate genes. The list is based primarily on the data from the QTLs boundaries (Supplementary Table 3). The list was filtered for non-synonymous polymorphism in the coding region of the genes between the F1 parents (Supplementary Table 4). The final list contains six genes with the “HIGH” impact variant score as analyzed by the SnpEff software. Based on annotation, the list contains, among others, several genes that are involved in sugar transport (gene ID: Prudu_004403, Prudu_004404, Prudu_004408; Supplementary Figure 3). Preliminary results demonstrated a high negative correlation in the F1 population between cork layer (i.e., periderm) development (qualitative 1–5 scale measurements) and the SPC (data not shown). These data suggest that periderm development genes may be involved. For example, *HXXXD*-*type acyl-transferase* and *MYB-family transcription factor* (gene ID: Prudu_018862, Prudu_018912; Supplementary Figure 3) genes were suggested to be involved in cork synthesis (Soler et al., 2007; Vulavala et al., 2019). Interestingly, the *EPIDERMAL PATTERNING FACTOR-like protein 2* (EPF2) gene is a member of this list (Supplementary Table 3; Prudu_018883). The EPF2 gene is a direct regulator of epidermis cell development and stomatal density (Hara et al., 2009). Although the EPF2 gene looks as a promising candidate to control SPC through its role in controlling stomatal density, advanced genetic research should done for establishing the link between the SPC phenotype and this gene. However, this study indicates that the variation of SPC trait in the F1 population is controlled by a small number of genes localized to only two loci in the almond genome. This work sets the stage for further studies aimed to delineate the genetic nature of the SPC trait, define its importance to the tree, and understand how it could be utilized for tree improvement targeted to produce fruit trees adapted to extreme climate. A recent study demonstrated that the cultivated almond breeding lines are highly conserved and are founded only on the cvs. Tuono, Cristomorto, and Nonpareil (Pérez de los Cobos et al., 2021). This study emphasizes the importance of introduction and utilization of genetic material originating from wild sources. Our current study sets the way for utilizing the wild almond *P. arabica* as a new source for widening and enriching the current narrow base of almond breeding material.

## Conclusion

This article is the first to establish a genetic study on mapping the unique SPC originating from the wild *P. arabica* almond. Here, we localized the genetic components that regulate the trait and narrowed the whole ∼240-Mb almond genome toward only two loci, with one major locus spanning only ∼400 kb and explaining 67% of the SPC phenotype, which eventually provided a list of 64 candidate genes. Forward genetic approach based on the establishment of a cross-bred population with genetic mapping and GWAS provides a remarkable infrastructure for future introduction of beneficial traits from wild almond origin. This approach is highly efficient for both studies of the genetics of important agricultural traits and introducing of new breeding material into highly conserved almond cultivars.

## Materials and Methods

### Plant Material

All trees are growing in the almond orchard in Newe Ya’ar Research Center in the Yizre’el Valley (latitude 34°42′N, longitude 35°11′E, Mediterranean temperate to subtropical climate). The parents of the F1 population, *P. arabica*, and the Israeli leading commercial cv. Um el Fahem (U.E.F.) are grown at two copies for each, grafted on GF-677 rootstock, and planted in winter 2018. The F1 population (*P. arabica* X U.E.F.) contains 92 seedlings that were germinated in the nursery in winter 2017 and replanted in the orchard in winter 2018.

### Gas Exchange Measurements

Gas exchange measurements were done in the field on 1-year-old stems (i.e., current year growth) of 3-year-old *P. arabica* and *P. dulcis* (U.E.F.) trees, from October 2019 to October 2020. Each month, 2 reciprocal days were chosen; in each day, four stems per genotype were analyzed (*n* = 8 per month). All measurements were taken between the hours 8:30 and 10:30 a.m. (the latest were in winter). When there were leaves on the stems, they were removed 2 days prior to measurements to eliminate wounding stress effect. Measurements were carried out with the LI-6800 Portable Photosynthesis System (LI-COR Biosciences, United States), using the 6 × 6-needle chamber, which is compatible with tree branches of 2.5–4.5 mm diameter. The following conditions were held constant in the chamber: photon flux density of 1,200 μmol m^–2^ s^–1^ (90% red, 10% blue) and CO_2_ reference of 400 PPM was set. Chamber relative humidity and the temperature were held for each month according to the multiannual average. Gas exchange results were normalized to stem surface area and displayed as net assimilation rates (μmol CO_2_ m^–2^ s^–1^), transpiration rates (mmol H_2_O m^–2^ s^–1^), and instantaneous water use efficiency (iWUE; the ratio between net assimilation and transpiration rates).

Gas exchange measurements on the F1 population were conducted in February 2020 for 2 weeks, while the trees were dormant, between the hours 9:30 and 11:30 a.m. In dormancy, there is no interaction with the photosynthetic state of the tree leaves, and the results are more stable. Four stems were measured (*n* = 4) for each genotype. The measurement protocol was the same as mentioned above. The measurements were conducted in 2 successive years, 2019 and 2020. Those of 2019 were conducted with a portable instrument (CIRAS 3 pp-system, United States). Nonetheless, they were significantly correlative with the results obtained with the Licor 6800 instrument in 2020 (correlation of 0.67, *P*-value < 0.0001 by Spearmen). To determine stem respiration rates, stem gas exchange measurements were conducted under the same conditions as described above. Next, the Licor 6800 light source was turned off for ∼2 min (for the stabilization of ΔCO_2_), and data were recorded. In dark, the net assimilation value represents respiration. Stem respiration rate was recorded under 17, 28, and 34°C.

### Whole-Genome Sequencing and SNP Calling

Using the plant/fungi DNA isolation kit (Norgen Biotek Corp., Canada), DNA extracted from young leaves. DNA of *P. arabica* and U.E.F. was sent to Macrogen (Macrogen, South Korea) for WGS—Illumina Nova Seq 6000, with a targeted coverage of X50 on average, read length of 150 bp with paired-end sequencing. OmicsBox software (version 1.3.11^3^) was used for preprocessing the raw reads based on Trimmomatic (Bolger et al., 2014) for removing adapters and contamination sequences, trimming low-quality bases, and filtering short and low-quality reads. The cleaned reads were mapped onto the reference genomes: *P. dulcis* cv. Lauranne (text foot note 2) and *P. dulcis* cv. Texas (text foot note 1), using the Burrows-Wheeler Aligner (BWA) software 0.7.12-r1039, with its default parameters (Li and Durbin, 2009). The resulting mapping files were processed using SAMtools/Picard tool^4^ (version 1.78) (Li et al., 2009), for adding read group information, sorting, marking duplicates, and indexing. Then, the local realignment process for locally realigning reads was performed so that the number of mismatching bases was minimized across all reads using the RealignerTargetCreator and IndelRealigner of the Genome Analysis Toolkit version 3.4-0 (GATK^5^) (Depristo et al., 2011). Finally, the variant calling procedure was performed using HaplotypeCaller of the GATK toolkit^6^ developed by Broad Institute of MIT and Harvard (Cambridge, MA, United States). Only sites with DP (read depth) higher than 20 were further analyzed. SnpEff program (Cingolani et al., 2012) was used to categorize the effects of the variants in the genomes (Supplementary Tables 1, 4). The program annotates the variants based on their genomic location (intron, exon, untranslated region, upstream, downstream, splice site, or intergenic regions), including in the Almond GFF file extracted from the NCBI database (GCA_008632915.2). Then, it predicts the coding effect such as synonymous or non-synonymous substitution, start or stop codon gains or losses, or frame shifts.

### Population Genotyping

Based on WGS of *P. arabica* and U.E.F., a SNP calling was performed in order to select SNPs that will detect polymorphism within the F1 population. The following criteria were set: (1) remove sites with DP lower than 20; (2) an isolated SNP over 100-bp interval; (3) the SNP is unique with no matching on other genomic regions on the reference genome; (4) informative SNPs for the F1 population that are homozygous for one parent and heterozygous for the other. In addition, SNPs were chosen at intervals of 40 kb along the almond genome (*P. dulcis* cv. Lauranne; text foot note 2) to obtain an unbiased representation through the whole chromosomes. Overall, a set of 5,000 markers was selected for genotyping (Figure 3). The F1 population screening was accomplished by “targeted SNP seq” by LGC (LGC Genomics, Germany) for SNP genotyping.

### Genetic Map Construction

For generating the genetic map, the JoinMap^®^ 4.1 software (Van Ooijen and Jansen, 2013) was used. CP population type was used with the code lmxll for markers that were homozygous for the male parents (*P. arabica*) and heterozygous in the female parent (U.E.F.), and the code nnxnp for the opposite case. Because there were no common markers (hkxhk), we did not combine the two marker types, and undertook the pseudo-test-cross method (Swinburne and Lindgren, 2013), meaning that we separated the markers into two different maps, one map for the U.E.F. (where *P. arabica* is homozygous-lmxll code) and one for the *P. arabica* parent (where U.E.F. is homozygous nnxnp code). Markers were filtered for three parameters: (1) more than ∼11% missing data, (2) non-Mendelian segregation (*X*^2^ > 6.5, DF = 1), and (3) remove markers in similarity of 1.0. The “Independence LOD” algorithm was used for LGs clustering (LOD > 8), and the Kosambi’s function was chosen for calculating genetic distance.

### Mapping of QTL

In order to conduct the QTL analysis, we used the Map QTL^®^5 software (Van Ooijen, 2006). QTLs and their significance were calculated using IM. A QTL was determined as significant when its LOD score was higher than the calculated threshold (1,000 permutation at α = 0.05), and the QTL spanning was determined by ±1 LOD from the max LOD marker.

### Genome-Wide Association Study

Association was calculated by TASSEL 5.2.59 (Bradbury et al., 2007). The set of SNPs was filtered; marker discarded when missing data was >8.6%, and the allele frequency was set for 0.2 < x < 0.8 for preventing overestimated impact of rare alleles. The general linear model (GLM) was applied for the phenotypic and genotypic intersect data set to test the association. Threshold for significance result was assessed by 1,000 permutation test α = 0.05.

### Statistics

All significance tests were done by the statistical software JMP (JMP^®^ PRO 15.0.0© 2019 SAS Institute Inc.), α = 0.05. To test significance when the variance was unequal, a simple *t*-test was used, and if it was equal, the pooled *t*-test ANOVA was performed. Tukey–Kremer’s test was used to analyze variance in the population when the distribution was normal, and the variance inside the groups was equal; when it was not equal or normal, Wilcoxon non-parametric test was used. Broad-sense heritability of the SPC was calculated on the F1 (full sibs) by the “Rsquare adj” value, given by a simple ANOVA test.

## Data Availability Statement

The original contributions presented in the study are included in the article/Supplementary Material, further inquiries can be directed to the corresponding author.

## Author Contributions

HB designed and conducted all experiments and wrote the manuscript. AD-F processed and assembled the row sequences of the parent’s DNA, the SNPs calling, analyzing the SNPs effect, and all genes’ annotation. IB-Y and RH-B generated the F1 population. TA-S, ZA, and HB developed the infrastructure for measuring stem gas exchange. DH designed the experiments, supervised the study, and wrote the manuscript. All authors discussed and commended on the manuscript.

## Conflict of Interest

The authors declare that the research was conducted in the absence of any commercial or financial relationships that could be construed as a potential conflict of interest.

## Publisher’s Note

All claims expressed in this article are solely those of the authors and do not necessarily represent those of their affiliated organizations, or those of the publisher, the editors and the reviewers. Any product that may be evaluated in this article, or claim that may be made by its manufacturer, is not guaranteed or endorsed by the publisher.

## Footnotes

1
https://www.ncbi.nlm.nih.gov/bioproject/572860

2
https://www.ncbi.nlm.nih.gov/bioproject/553424

3
https://www.biobam.com/omicsbox/

4
http://broadinstitute.github.io/picard/

5
http://www.broadinstitute.org/gatk/

6
https://gatk.broadinstitute.org/hc/en-us
