# Supplementary material for: Revealing the Genetic Components Responsible for the Unique Photosynthetic Stem Capability of the Wild Almond Prunus arabica (Olivier) Meikle
Source: Front Plant Sci. 2021 Nov 25;12:779970. doi: 10.3389/fpls.2021.779970 (PMC8657148; doi:10.3389/fpls.2021.779970)
Supplement: Supplementary file 1 [file Data_Sheet_1.docx]

Supplementary Material

*Supplementary Tables S1-S4 (see separate file for Supplementary Table S5)*

**Table S1. Variant’s effect.** Summarized variant’s (SNPs and InDels) effect comparing between *P. arabica* and U.E.F. All data shown analyzed by the SnpEff program (SnpEff 5.0d version). Only SNPs with DP>20 were analyzed.


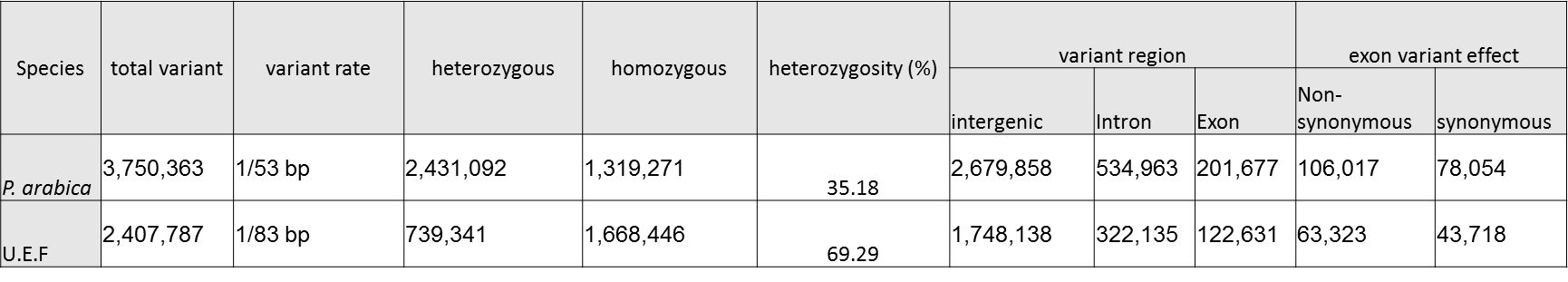


**Table S2**. Genotyping quality parameters for the selected SNPs. Data provided by LGC Genomics (LGC Genomics, Germany).


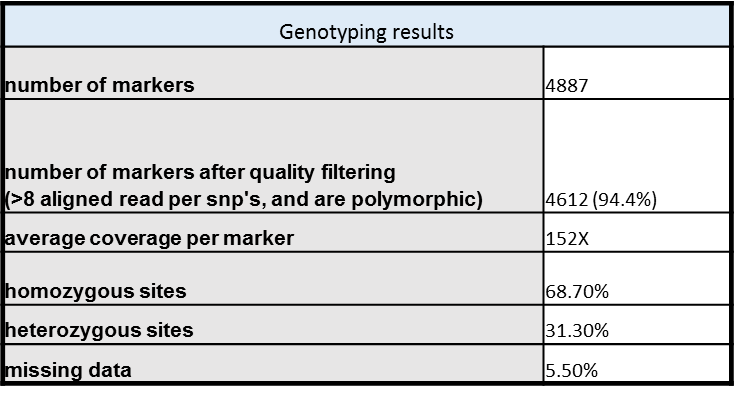


**Table S3. List of highly linked markers, detected by GWAS and QTL mapping, regulating the SPC trait.** Markers highly associated with the QTLs are presented according to their method of detection. Grey markers represent markers from chr-0 which were found by the genetic map on chr-7. The QTL boundaries defined by ±1 LOD or by the significance threshold. Marker ID also represents its physical position.


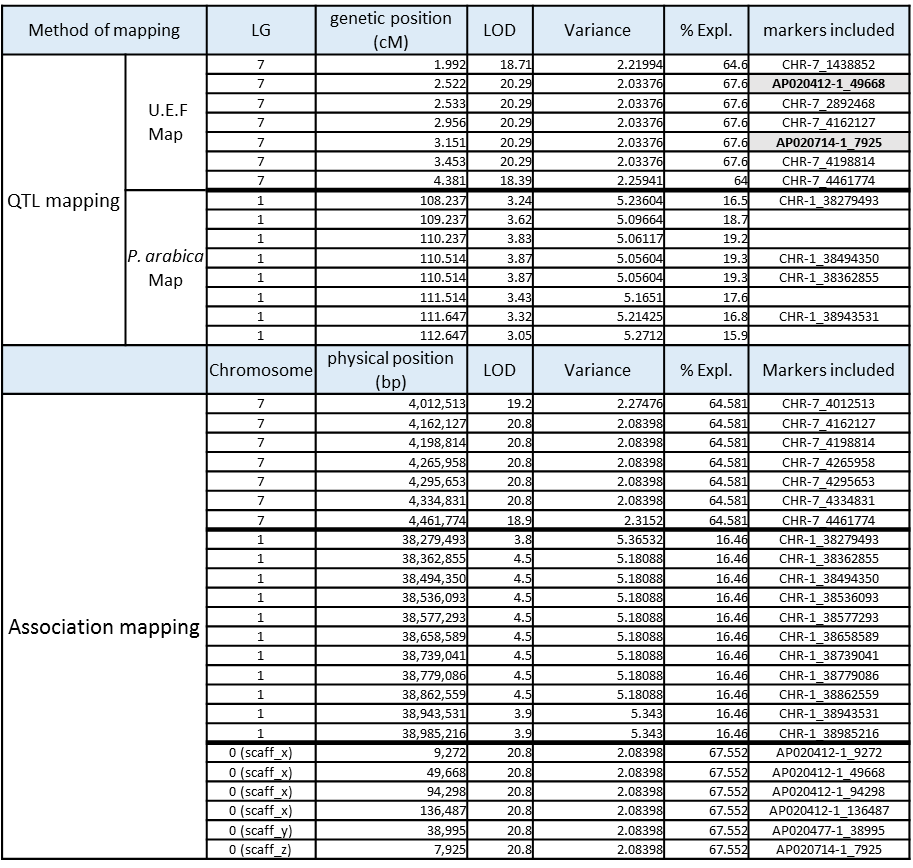

**Table S4. Candidate genes annotation list.** Annotation of the genes located in the DNA region underlined by the two QTL’s according to their physical position, after filtering for genes with only non-synonymous variant located in the coding region. The orange rows represent genes from chr-0 that were genetically mapped to Locus 7.
